# Research progress on the correlation between blood pressure variability and hypertensive microvascular disease

**DOI:** 10.3389/fcvm.2026.1759344

**Published:** 2026-02-09

**Authors:** Shifeng Chen, Hongyu Kuang, Jie Zhu, Jia Wei, Pei Pan, Xinyu Hu, Ke Yang, Xiaoshu Yi, Huaan Du

**Affiliations:** Department of Cardiology, University-town Hospital of Chongqing Medical University, Chongqing, China

**Keywords:** ambulatory blood pressure monitoring, blood pressure variability, hypertension, hypertensive microvascular disease, target organ damage

## Abstract

The hypertensive microvascular disorder is a significant complication that can lead to substantial target organ damages, including cognitive impairments, visual impairments, and deterioration in renal function. Recent studies have indicated that blood pressure variability (BPV) is an independent risk factor for the progression of this pathology. The present paper aims to systematically elucidate the concept, classification, and clinical significance of BPV, focusing on how it acts as a pathogenic mechanism independent of mean blood pressure to exacerbate endothelial injury and cause target organ damage. This review was conducted to evaluate the influences of multiple factors on BPV, including neurohumoral regulation, the behavioural environment and comorbidities. It also emphasises the intrinsic link between BPV and microvascular complication risk in specific populations, such as those with diabetes and obesity. In summary, it is evident that a comprehensive exploration of the underlying mechanisms of BPV is imperative for the early prevention and treatment of hypertensive microvascular diseases.

## 1 Introduction

Hypertension is one of the most prevalent chronic cardiovascular diseases globally. An epidemiological research suggests that approximately 1.4 billion adults worldwide will be living with hypertension by 2024. However, it is estimated that less than one-fifth of these individuals will achieve adequate control of their condition. This phenomenon has been shown to result in a significant health and economic burden, particularly severe in developing countries (1). It is well established that persistent elevated arterial blood pressure can result in damage to multiple target organs, including but not limited to the heart, brain, and kidneys. This has a considerable impact on quality of life and increases healthcare costs, making effective blood pressure control crucial for mitigating target organ damage and improving prognosis. It is widely accepted that microvasculature represents a key target organ for damage caused by hypertension. This term refers to a number of different organs and arteries, including those located within the brain (intracranial arterioles), the eye (retinal arteries), the kidneys (glomerular arterioles), and the heart (coronary arteries). Early microvascular injury is characterised by its subtlety and difficulty in detection, with a progressive deterioration that accompanies disease progression. This contributes to a range of serious conditions, including stroke, cognitive impairment, visual impairment, coronary atherosclerotic heart disease, and renal insufficiency. At present, there is no universally accepted standard for the detection of microvascular lesions, thus highlighting the necessity for further exploration of innovative diagnostic approaches.

Blood pressure variability (BPV) is a term used to describe a series of indicators reflecting fluctuations in systolic or diastolic pressure. Notwithstanding mean blood pressure levels, BPV evinces a marked association with cardiovascular incidents (2). The key indicators for assessing BPV include standard deviation (SD), coefficient of variation (CV), average real variance (ARV), and variance independent of mean (VIM). Despite the differing calculation methods, elevated values across these metrics generally indicate increased blood pressure fluctuations. BPV is recognised as an independent risk factor for microvascular complications in hypertension and has a promoting effect on microvascular target organ damage (3–6). Maintaining BPV stability may therefore be crucial. To date, no study has systematically elucidated the role of BPV in microvascular complications across different hypertensive populations or its underlying mechanisms. The present review explores the correlation between BPV and microvascular complications, and investigates potential mechanisms.

## 2 An overview of BPV

### 2.1 The definition and classification of BPV

BPV is defined as the degree of blood pressure fluctuation exhibited by an individual over a specific time period. This fluctuation is indicative of the body's ability to regulate blood pressure in response to internal and external environmental changes, as well as the integrity of cardiovascular autonomic nervous system regulation. While healthy individuals also experience a certain range of blood pressure fluctuations during daily activities, the amplitude of these fluctuations is minimal and remains within a controllable range. However, in cases where the body is in a diseased state, such as hypertension, atherosclerosis, or diabetes, there is often an accompanying autonomic dysfunction and a decline in the function of vascular pressure receptors. This directly leads to a decline in the body's ability to regulate blood pressure both instantaneously and over the short term, resulting in an abnormal increase in BPV (7–9). Consequently, BPV abnormalities are fundamentally the combined result of autonomic nervous system dysfunction and impaired baroreceptor reflexes. Clinically, BPV can be categorized based on the time span of blood pressure monitoring into ultra-short-term BPV (beat to beat), short-term BPV (within 24 h), medium-term BPV (within several days), and long-term BPV (spanning weeks, seasons, or even years) (10). Short-term BPV refers to blood pressure fluctuations over 24 h, as assessed by ambulatory blood pressure monitoring (ABPM). This includes daytime BPV, nocturnal BPV and the morning blood pressure surge, which is the most clinically prevalent form (11). Medium- and long-term BPV reflect blood pressure changes over a period of days to years and are usually associated with BPV between follow-up visits. These exhibit independent predictive value for events such as myocardial infarction, stroke, and cardiovascular mortality (12–14). Ultra-short-term BPV (beat-to-beat) represents blood pressure fluctuations between consecutive heartbeats and can be obtained via invasive arterial monitoring or non-invasive devices (15, 16). It is recognised as an independent predictor of white matter lesions, recurrent stroke and cardiovascular events (17, 18), and is valuable in assessing fluid responsiveness in mechanically ventilated intensive care patients (19) (Table 1).

**Table 1:** Definitions, assessment methods, indicators, physiological and pathological significance, clinical value, advantages, and limitations of different types of BPV.

Type of BPV	time scale	Monitoring methodology	Representative indicators	Physiological/pathological significance	Clinical association	Advantages/limitations
Ultra-short-term BPV	beat to beat	Invasive intra-arterial monitoring, Non-invasive photoplethysmography, pulse transit time, tonometry	Linear: SD, CV, ARV, VIM, RSD, SV	Reflects instantaneous changes in the cardiovascular autonomic nervous system and is highly correlated with baroreflex sensitivity	Characteristic analysis in hypertensive populations; predicts white matter lesions, recurrent stroke, cardiovascular events; perioperative risk assessment for cardiac surgery; evaluation of volume responsiveness in critically ill patients	Advantage: Captures real-time autonomic nervous system information, reflecting early pathological changes. Limitation: Subject to equipment and environmental constraints; operation is complex; not yet widely adopted in clinical practice
			Nonlinear: measure of entropy, DFA			
Short-term BPV	in 24 h/day/night	ABPM	24-hour/daytime/nighttime SD, CV, ARV, VIM, nocturnal blood pressure drop, morning peak blood pressure, etc	Reflects the impact of circadian rhythm, behavioral activities, and sleep patterns on blood pressure, demonstrating short-term vascular regulatory capacity	Independently predicts risk of coronary heart disease, stroke, and cardiovascular mortality; used in evaluate antihypertensive treatment efficacy	Advantage: Relatively high degree of standardization; most widely used in clinical practice; evidence is relatively robust
						Limitation: Only reflects single-day blood pressure variation; heavily influenced by sleep and physical activity
Medium-term BPV	day to day	OBPM/HBPM	SD, CV, ARV, VIM, etc	Reflects blood pressure stability over longer periods, influenced by treatment adherence and lifestyle fluctuations	Predict cardiovascular mortality risk	Advantage: Data easily obtained (based on routine follow-up), low cost. Limitation: Susceptible to “white-coat effect” and inconsistent measurement conditions
Long-term BPV	visit to visit	Office, home, or ABPM data from long-term follow-up	SD, CV, ARV, VIM, etc	Reflects long-term trends and chronic instability of blood pressure, associated with progressive vascular remodeling and target organ damage	Predicts stroke, cognitive impairment, renal progression; predict mortality risk in CVD patients; assesses adherence, aids medication decisions	Advantage: Capable of capturing blood pressure fluctuation patterns most relevant to chronic damage
						Limitation: Requires very long-term data; analysis is complex; many confounding factors

BPV, blood pressure variability; SD, standard deviation; CV, coefficient of variation; ARV, average real variability; VIM, variability independent of the mean; RSD, residual successive difference; SV, successive variance; DFA, detrended fluctuation analysis; ABPM, ambulatory blood pressure monitoring; OBPM, office blood pressure measurements; HBPM, home blood pressure monitoring; CVD, cardiovascular disease.

### 2.2 Multiple factors greatly influencing BPV

The current body of research indicates that BPV is primarily influenced by multiple factors, including the regulation of the autonomic and humoral systems is of particular interest in this study. The autonomic nervous system plays a dominant role in maintaining blood pressure stability, with the dynamic balance between sympathetic and parasympathetic activity determining BPV levels (20). Dysfunction of this system, such as cardiovascular autonomic neuropathy associated with diabetes, has been demonstrated to significantly reduce baroreflex sensitivity and lead to elevated blood pressure (21). Furthermore, the circadian secretion patterns of endocrine hormones such as aldosterone, cortisol, and thyroid hormones have been identified as significant contributors to abnormal BPV (22). Secondly, the behavioural and environmental factors must be considered. Numerous elements of daily life have been demonstrated to exert an influence on BPV, including emotional fluctuations, sleep quality, seasonal changes, dietary patterns, and exercise habits (23–28). Thirdly, consideration must be given to the combined effects of medications and other diseases. The types and combinations of antihypertensive medications are significant factors influencing BPV (29). Furthermore, the omorbid conditions of patients are also significant contributors to BPV fluctuations, particularly in the diabetic population, where obesity, kidney disease, and sleep-disordered breathing often coexist (30–32). The presence of these comorbid states has been demonstrated to exacerbate BPV abnormalities.

### 2.3 Pathophysiological mechanisms of BPV-induced endothelial dysfunction and microvascular injury

Research indicates that BPV contributes to vascular endothelial injury and microvascular lesions via multiple pathophysiological mechanisms. These include: 1. Abnormal blood flow shear stress. Increased BPV causes abrupt and recurrent changes in blood flow velocity and direction, generating turbulent and oscillatory shear stress. This directly damages vascular endothelial cells, leading to reduced nitric oxide (NO) bioavailability and promoting reactive oxygen species (ROS) production. It also activates inflammatory pathways such as nuclear factor kappaB (NF-*κ*B) (33, 34). 2. Vascular remodelling: Persistent abnormal mechanical stress stimulates the proliferation, migration of vascular smooth muscle cells and extracellular matrix deposition. This leads to arterial wall thickening and luminal narrowing (35). 3. Promotion of thrombogenesis: Under conditions of high shear stress, endothelial injury exposes subendothelial collagen, which triggers von Willebrand factor (vWF)-mediated platelet activation and promotes microthrombus formation (36).

## 3 BPV also acting as an independent risk factor for hypertensive microvascular complications

### 3.1 Hypertensive retinopathy

Hypertensive retinopathy (HR) is a prevalent microvascular complication of hypertension, manifesting as lesions in various locations, including the retina, choroid, and optic nerve. A multicentre cross-sectional study involving 696 adult hypertensive patients revealed an overall prevalence of HR reaching 57.47% (95% CI: 53.75–61.10) (37). Conventionally, the progression of hypertension has been closely associated with the extent of blood pressure elevation. In the early stages of elevated blood pressure, functional compensatory adjustments occur in retinal arterioles, leading to diffuse stenosis. This progresses structurally over time, manifesting as retinal arteriolar wall thickening and hyalinization, ultimately compromising the blood-retinal barrier and precipitating HR (38). In recent years, the role of BPV in HR development has garnered increasing attention from the academic community, though current research conclusions remain inconclusive. A substantial body of research has indicated a positive correlation between BPV and HR. Previous studies also evaluated the association between BPV metrics and retinal microvascular structural degeneration in hypertensive patients (39). These findings demonstrated a negative correlation between SD of 24-hour diastolic blood pressure and retinal nerve fibre layer thickness (*β* = −0.707, *p* = 0.002). In addition, it was found that systolic blood pressure average real variability(ARV) was negatively correlated with vessel density (VD) (*β* = −0.259, *p* = 0.001) and perfusion density (PD) (*β* = −0.006, *p* = 0.001). Then, Lou et al. (40) demonstrated in a diabetic cohort that elevated systolic BPV and systolic BP > 130 mmHg were both independent risk factors for retinopathy. In a large-scale study enrolling patients with type 1 diabetes, Fatulla et al. (41) identified inter-visit systolic blood pressure variability (SBPV) as an independent predictor of retinopathy. Meanwhile, the highest quartile of inter-visit SBP variability was significantly demonstrated to be associated with a 17% increased risk of new-onset retinopathy (OR = 1.171, 95% CI: 1.129–1.214, *p* < 0.001) (42). However, a number of well-designed, large-scale studies have failed to replicate these findings, and in some cases have even reported clear negative results. Foo et al. (43) found in an Asian type 2 diabetic cohort that factors associated with moderate retinopathy included mean glycated hemoglobin (HbA1c) levels and mean systolic blood pressure, but not BPV. Cardoso et al. (44) proposed that BPV is a predictor of major adverse cardiovascular events; however, they also found that it is unrelated to various microvascular outcomes, including retinopathy. This discrepancy may be attributable to two factors. Firstly, the underlying disease types of the study populations. Positive results predominantly emerged in primary hypertension cohorts, while negative findings were common in type 2 diabetes populations due to strong interference from multiple metabolic factors, such as hyperglycaemia. Secondly, a considerable degree of heterogeneity exists among the studies in terms of the assessment metrics for BPV, temporal scales, and diagnostic criteria for retinopathy, which complicates the direct comparison of results. In summary, the current evidence remains insufficient to establish a universally applicable causal relationship between BPV and retinopathy. In order to achieve further validation, it is necessary to conduct future large-scale prospective studies employing standardised assessment systems across stratified populations.

### 3.2 Coronary heart disease

Coronary heart disease (CHD) has been identified as the primary cause of cardiac death among hypertensive patients. Concurrently, BPV is considered a potential independent risk factor for the onset and progression of CHD (12, 45, 46). D. Clark et al. (12) established a correlation between long-term systolic blood pressure (SBP) and the progression of coronary atherosclerotic plaques (OR = 1.09, 95% CI: 1.02–1.17, *p* = 0.02). Moreover, the relationship between BPV and mortality was analyzed, indicating that patients in the highest quintile of BPV over a one-year period exhibited a significantly elevated long-term mortality risk in comparison to those with the lowest BPV (45). It remained consistent across different methods of measuring systolic BPV, with the strongest association observed when expressed as ARV (aHR = 1.18, 95% CI: 1.08–1.30). As demonstrated by Sun PF et al. (46), a U-shaped relationship between short-term 24-hour systolic BPV and one-year mortality in CHD patients (*p* = 0.001), while diastolic BPV showed a positive correlation (HR = 1.03, 95% CI: 1.00–1.06). Consequently, elevated BPV—whether short-term or long-term—is associated with adverse outcomes in CHD patients, with systolic BP demonstrating a stronger correlation. Furthermore, data from the ACSOT-BPLA trial indicate that antihypertensive regimens including amlodipine may improve BPV and potentially reduce the risk of stroke, coronary events, and cardiovascular mortality (29, 47). The potential benefits of this phenomenon may be attributable to drug-induced peripheral vasodilation, altered arterial compliance, and reduced circulatory load. It is evident that coronary plaque rupture and thrombosis are significant contributors to the progression of CHD. Therefore, the reduction of BPV in hypertensive patients has the potential to decrease vascular shear stress and suppress inflammatory mediators, such as C-reactive protein. This, in turn, has been demonstrated to reduce subendothelial lipid deposition, thereby slowing atherosclerosis progression and decreasing adverse cardiovascular events (4).

### 3.3 Stroke

A substantial body of epidemiological research has indicated that as many as 73.91% of stroke patients also have hypertension (48). Stroke represents the primary cause of cognitive impairment, disability, or death among hypertensive patients. Furthermore, BPV has been found to be closely associated with the occurrence and progression of stroke, as well as with white matter ischemic damage and cognitive impairment (49). Research findings indicate a robust pathophysiological association between BPV and cerebral microvascular injury. Abnormal BPV has been demonstrated to induce significant fluctuations in blood pressure, directly contributing to the occurrence of arterial ruptures or occlusions, and consequently leading to the manifestation of stroke (5). Conversely, elevated BPV has been shown to augment shear stress on vascular walls, a process that may result in microvascular dysregulation and arterial remodelling through the infliction of direct damage to endothelial cells (50). Concurrently, abnormal vascular wall shear stress has been demonstrated to further expose subendothelial collagen and trigger vWF-mediated platelet activation, thereby promoting thrombosis (36). Research indicates that short-term BPV elevation is a potent stroke trigger, such as morning blood pressure surge (MBPS), which has been associated with stroke risk in patients with Big Dipper hypertension (aHR = 1.14, 95% CI: 1.00–1.30) (51). Heshmatollah et al. (13) conducted a prospective analysis of 9,958 stroke-free subjects, revealing that long-term BPV is closely associated with stroke occurrence, with systolic BPV showing the strongest correlation with haemorrhagic stroke (HR = 1.27, 95% CI: 1.05–1.54). Furthermore, persistent BPV abnormalities have been found to correlate with white matter ischemic lesions, cognitive decline, and widespread structural alterations in cerebral white matter (52, 53). Consequently, BPV has been demonstrated to play a pivotal role in both chronic cerebrovascular injury and acute stroke events. It is evident that substantial fluctuations in blood pressure have the capacity to induce arterial rupture in a direct manner. Moreover, heightened vascular wall shear stress has been observed to result in endothelial injury and the promotion of thrombosis. Improving BPV in hypertensive patients may therefore hold clinical value for preventing microvascular brain injury.

### 3.4 Hypertensive renal disease

Hypertensive renal disease (HRD) is a prevalent form of chronic kidney disease (CKD) and a principal cause of end-stage renal disease (ESRD) (54). The demographic shift towards an ageing population, coupled with the escalating prevalence of hypertension, portends a relentless rise in the incidence and mortality rates of HRD. This augurs well for the challenges that kidney disease prevention and management face. Patients with HRD frequently exhibit elevated blood pressure levels, and the onset and progression of this condition is often attributed to inadequate blood pressure control. Nevertheless, this standpoint has been contested by the principles of evidence-based medicine. The Systolic Blood Pressure Intervention Trial (SPRINT) demonstrated that while intensive blood pressure lowering reduced cardiovascular events, it did not yield equivalent improvements in renal outcomes (55). This paradox suggests that blood pressure levels may not be the primary determinant of renal prognosis. In recent years, the scientific community has increasingly recognised the critical role played by BPV. It is important to note that the current research focus has shifted from static BPV at a single time point to its dynamic evolution over several years, which is essential for assessing long-term risk. Cheng et al. (56) provided the first systematic demonstration that increased systolic BPV during follow-up is an independent risk factor for new-onset chronic kidney disease and renal function deterioration (aHR = 1.33, 95% CI: 1.15–1.52). This indicates a strong association between BPV and renal disease prognosis. The mechanisms by which BPV promotes HRD may involve multiple pathways, including abnormal shear stress fluctuations, vascular remodeling, endothelial dysfunction, and activation of the renin-angiotensin-aldosterone system (57). The urine albumin-to-creatinine ratio (UACR) and estimated glomerular filtration rate (eGFR) are pivotal indicators of renal function, closely associated with cardiovascular disease and CKD prognosis. As demonstrated by Wang et al. (58), there is a strong association between BPV and new-onset CKD and albuminuria in hypertensive patients, with systolic BPV demonstrating a more pronounced relationship. In comparison with the lowest quartile, the highest quartile of systolic BPV was associated with a 90% increased risk of CKD (aHR = 1.90, 95% CI: 1.13–3.19), while the risk of albuminuria events increased by 67% (aHR = 1.67, 95% CI: 1.14–2.45). A prospective study (59) also indicated that long-term increased BPV correlates with albuminuria and declining eGFR. The evidence presented herein indicates that elevated and worsening BPV trends constitute independent risk factors for HRD onset and progression. The pressing question that must be addressed is: The present study seeks to investigate whether treatment strategies that specifically target BPV reduction, in addition to standard antihypertensive therapy, can further improve outcomes in patients with HRD. This finding may indicate a promising avenue for future prospective randomised controlled trials.

## 4 BPV promoting microvascular damages in different populations

### 4.1 Metabolic syndrome population

Metabolism is currently recognised as a key factor influencing microvascular disease. Consequently, patients exhibiting diverse metabolic profiles (e.g., diabetes, obesity) demonstrate varying sensitivities and stability to BPV (30, 60–66). The identification of BPV differences across distinct disease states facilitates precise assessment of microvascular disease risk and improves prognosis.

Prolonged hyperglycaemia and blood glucose fluctuations experienced by diabetic patients have been demonstrated to result in oxidative stress, chronic inflammation, and the accumulation of advanced glycation end-products (AGEs). These phenomena have been shown to ultimately lead to endothelial injury and microvascular thrombosis (67). Consequently, diabetic patients are considered to be a high-risk group with regard to microvascular complications. Research has indicated that persistent fluctuations in blood pressure can promote inflammatory responses and exacerbate endothelial damage, thus establishing them as a pivotal factor in the development of diabetic target organ damage (68). A prospective study by Chen et al. (69) further indicated that baseline systolic BPV independently predicts the risk of developing diabetes (HR = 1.973, 95% CI: 1.333–2.920). A large meta-analysis confirmed that in type 2 diabetes patients, each one-standard-deviation increase in long-term systolic BPV significantly elevated the risk of microvascular complications (pooled HR = 1.12, 95% CI: 1.01–1.24) (61). This finding lends further support to the hypothesis that BPV may act as a crucial catalyst for the onset and progression of diabetes. Concurrently, recent studies indicate that SGLT-2 inhibitors effectively improve abnormal nocturnal blood pressure rhythms in diabetic patients by reducing BPV, maintaining blood pressure stability, and lowering the risk of adverse cardiovascular events (70). Furthermore, a prospective study indicated that intensive statin therapy (target LDL-C < 70 mg/dL) has been demonstrated to have significant cardiovascular protective effects, but only in diabetic patients with high BPV (63). This underscores the considerable potential of stratified treatment based on BPV levels for cardiovascular protection in diabetic patients.

Obesity has been identified as an independent risk factor for cardiovascular disease (71). The epidemiological evidence indicates that approximately 8% of hypertensive patients also have obesity (72), with this subgroup exhibiting more pronounced blood pressure fluctuations (73). Concurrently, studies indicate that obese patients face markedly increased risks of microvascular target organ damage, including retinopathy, coronary heart disease, and renal insufficiency (74, 75). Emerging evidence suggests that obesity may exacerbate microvascular target organ damage by increasing blood pressure instability through elevated BPV. Specifically, on one hand, the nocturnal obstructive sleep apnoea that is commonly associated with obesity can lead to excessive sympathetic nervous system activation. Conversely, as articulated by Koenen et al. (65), aberrant proliferation and remodelling of visceral adipose tissue precipitate heightened immune cell infiltration and secretion of multiple vasoconstrictive mediators, concomitant with diminished secretion of salutary vasodilatory factors such as adiponectin. The combined effect of these mechanisms is the disruption of the body's physiological regulation of blood pressure. A domestic cross-sectional study provides more direct epidemiological evidence: compared with individuals of normal weight, overweight individuals had a 10% increased risk of developing sustained systolic blood pressure (SBP) (HR = 1.10, 95% CI: 1.04–1.15), while this risk further escalated to 23% in obese individuals (HR = 1.23, 95% CI: 1.15–1.32) (30). It is noteworthy that recent scholarly contributions propose adipocyte dysfunction as a pivotal contributor to hypertension and abnormal BPV (66). Consequently, obese patients may exhibit higher BPV, and meticulous monitoring and control of blood pressure stability in obese patients can effectively reduce the risk of cardiovascular disease.

### 4.2 Elderly population

In addition to individuals with diabetes and obesity, the elderly are another high-risk group for developing microvascular complications. Aging itself increases the prevalence of hypertension in the elderly through multiple pathophysiological mechanisms, including atherosclerosis, decreased baroreflex sensitivity, altered renin-angiotensin-aldosterone system (RAAS) regulation and reduced renal sodium excretion (76). It also serves as a common basis for both short-term and long-term abnormal BPV elevation in this population. Elevated BPV places greater strain on microvascular beds that are already affected by vascular stiffening and impaired blood pressure self-regulation. This exacerbates endothelial injury, promotes inflammatory responses and vascular remodelling, and increases the risk of target organ damage independently of blood pressure levels. Recent studies indicate that elevated BPV is a strong predictor of cognitive impairment, dementia and cardiovascular events in older adults (77, 78). For example, the S.AGES prospective cohort study showed that inter-visit BPV is significantly correlated with an increased risk of cognitive impairment and dementia in people aged 65 years and over (aHR > 1.21, *p* < 0.029) (77). A *post-hoc* analysis of the ASPREE (Aspirin in Reducing Events in the Elderly) trial also confirmed that elevated blood pressure variability (BPV) during clinic visits was independently associated with an increased risk of ischaemic stroke (highest vs. lowest tertile: aHR = 1.56, 95% CI: 1.04–2.33) and cardiovascular disease (highest vs. lowest tertile: aHR = 1.36, 95% CI: 1.08–1.70) (78). It is noteworthy that the relationship between BPV and renal function varies by population. Further analysis of the ASPREE trial revealed that long-term BPV was unable to predict renal outcomes in the general elderly population (79), whereas markedly elevated BPV has been confirmed as an independent risk factor that accelerates renal deterioration in elderly patients with chronic kidney disease (80). Therefore, BPV should be considered as a target for antihypertensive treatment in elderly individuals with cardiovascular risk factors. The conflicting aspects of blood pressure management strategies in the elderly population must be recognised: sustained blood pressure reduction is necessary to minimise target organ damage, but excessive blood pressure lowering and inappropriate medication use may lead to cerebral hypoperfusion and orthostatic hypotension, increasing the risk of syncope, falls and cardiovascular events (81). Therefore, antihypertensive therapy for elderly patients should prioritise long-acting, stable antihypertensive agents (such as calcium channel blockers or renin–angiotensin system inhibitors) (82). Using home and ambulatory blood pressure monitoring to accurately assess circadian rhythms and amplitude of fluctuation is essential for reducing blood pressure variability in elderly patients and minimising microvascular target organ damage.

## 5 BPV-targeted interventions for microvascular protection

Blood pressure variability (BPV) is an independent predictor of cardiovascular events, distinct from mean blood pressure. It is strongly associated with microvascular damage and cardiovascular events. Therefore, it is essential to incorporate BPV into hypertension management strategies. An effective BPV intervention must combine individualised pharmacotherapy with systematic lifestyle modifications to stabilise haemodynamics through multiple pathways and to prevent microvascular injury.

Firstly, different antihypertensive drugs directly influence BPV. Studies indicate that treatment regimens incorporating long-acting dihydropyridine calcium channel blockers (such as amlodipine) or diuretics are more effective at reducing both short-term and long-term BPV (47, 83). De la Sierra et al. (47) concluded that amlodipine combined with diuretics is more effective than other major classes of antihypertensive drug at reducing short-term BPV in hypertensive patients, and this remains the case in combination therapy regimens. A *post hoc* analysis of the SPRINT trial further confirmed that sustained amlodipine therapy reduced systolic BPV by approximately 2.05 mmHg during follow-up (83), potentially due to its potent and sustained vasodilatory effects. Therefore, for patients with markedly elevated BPV, prioritising such agents in antihypertensive regimens may reduce residual cardiovascular risk.

Secondly, dietary intervention is a key strategy for stabilising BPV. It is crucial to restrict sodium intake and increase potassium consumption. Studies by Chang et al. (26) suggest that a lower urinary sodium-to-potassium ratio is associated with reduced short-term BPV. Specifically, the DASH diet alone has limited impact on reducing short-term BPV. However, combining the DASH diet with strict sodium restriction is a more effective way of lowering both blood pressure and short-term BPV. This highlights the importance of optimising dietary composition and strictly controlling sodium intake. Furthermore, increasing dietary nitrate intake can stabilise blood pressure by improving endothelial function and nitric oxide bioavailability. A randomised controlled trial confirmed that seven days of dietary nitrate supplementation significantly reduced blood pressure fluctuations and cerebral blood flow velocity in patients with transient ischaemic attacks (84). Therefore, incorporating evidence-based dietary modifications into hypertension management is a key strategy for controlling blood pressure and reducing BPV.

Finally, non-pharmacological approaches, such as regular exercise and weight management, can improve BPV. Meta-analyses indicate that regular exercise significantly improves both systolic and diastolic BPV, with aerobic exercise being particularly effective at reducing diastolic BPV (85). A randomised controlled trial demonstrated that a combination of aerobic exercise and resistance training is more effective than aerobic exercise alone in reducing short-term BPV (86). Additionally, proactive weight management is crucial for obese patients. A randomised controlled trial involving 100 obese, hypertensive patients demonstrated that adding bariatric surgery to baseline antihypertensive therapy reduced 24-hour BPV without altering the circadian rhythm of blood pressure (87). These findings further emphasise the importance of exercise and weight management for hypertensive patients with severe obesity in achieving target blood pressure levels.

## 6 Conclusions

**Figure 1:**
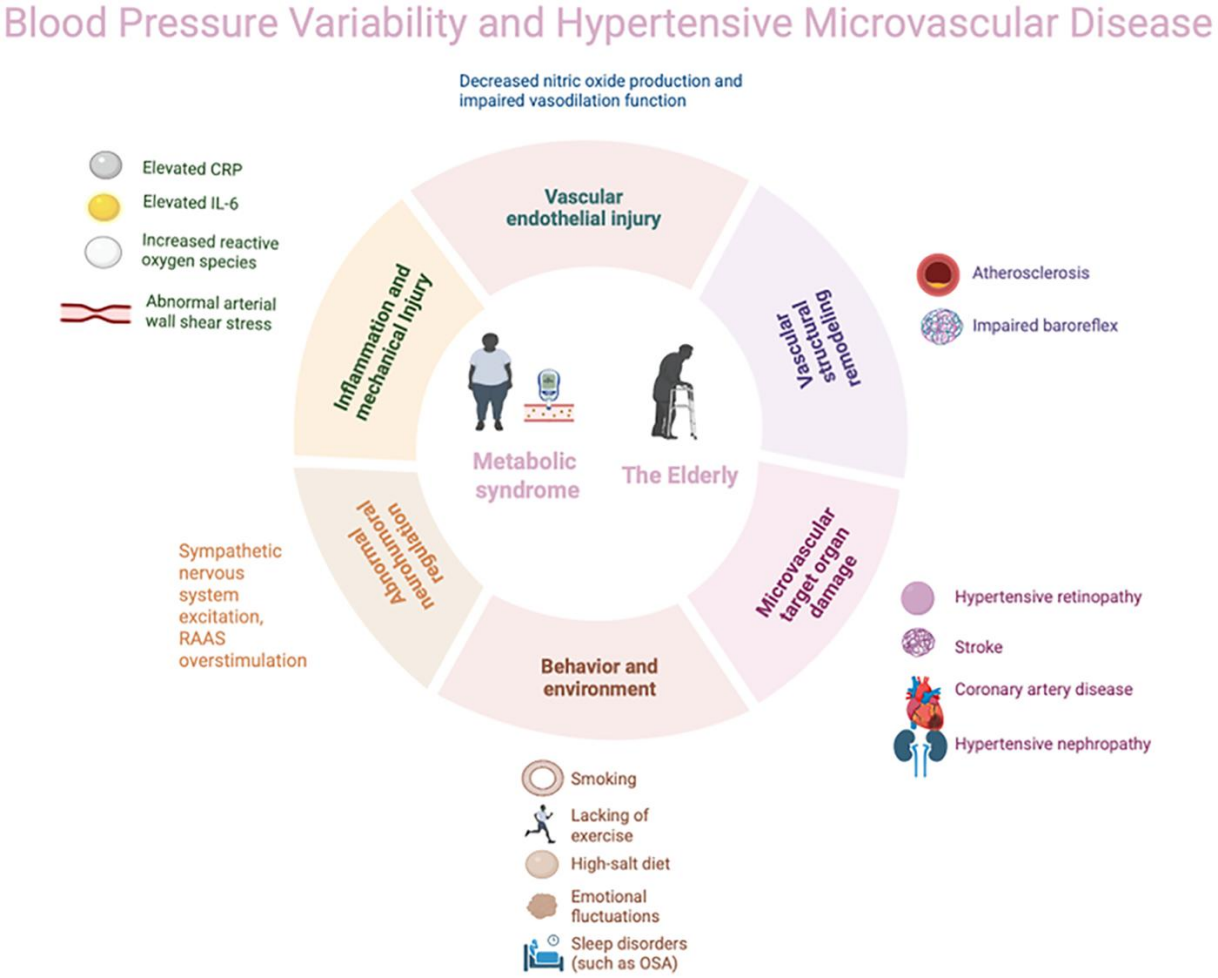
Blood pressure variability (BPV) and hypertensive microvascular disease.

In conclusion, blood pressure variability (BPV) has been identified as an independent risk factor for microvascular complications arising from hypertension and cardiovascular events, thereby emphasising its critical importance in the management of blood pressure (Figure 1). This current review has demonstrated that BPV exacerbates target organ damage independently of mean blood pressure levels through mechanisms such as aggravated endothelial injury and abnormal vascular shear stress. This is particularly evident in elderly populations and those with hypertension complicated by diabetes and obesity, also known as metabolic syndrome. Actually, the intrinsic link between BPV and microvascular complication risk is more complex, offering new perspectives for precision risk management. In clinical practice, beyond strict control of mean blood pressure, long-term monitoring and intervention targeting BPV should be integrated into hypertension management strategies. Ambulatory blood pressure monitoring (ABPM) and home blood pressure monitoring (HBPM) have been shown to be effective tools for the assessment of BPV. It is recommended that these techniques be adopted more widely in clinical settings in order to optimise blood pressure management and improve patient outcomes. Despite substantial research supporting BPV as an independent risk factor for microvascular damage in hypertension, there are currently significant limitations in this field that hinder its clinical translation. On the one hand, existing studies exhibit high heterogeneity, which is manifested in the following ways: (1) inconsistent methodological standards, including monitoring techniques (e.g., office blood pressure, ambulatory blood pressure monitoring and continuous monitoring), analytical indicators (e.g., SD, CV and ARV) and calculation methods; (2) varied temporal scales of focus (e.g., beat to beat, 24-hour and inter-visit), each carrying distinct physiological and pathological implications; and (3) substantial variations in the characteristics of the study population (e.g., age and comorbidities), which limits the generalisability of the findings. On the other hand, the most critical issue is the current lack of large-scale, prospective, randomized controlled trials demonstrating whether interventions specifically targeting BPV reduction can independently reduce microvascular complications and clinical events beyond the effects of lowering mean blood pressure levels. Therefore, future research urgently requires standardised BPV assessment protocols and intervention studies targeting clinical endpoints in clearly defined high-risk subgroups (e.g., patients with diabetes, obesity or advanced age), in order to advance the transition of BPV from a risk marker to a clinical therapeutic target.

## References

[1] Farrar J Frieden T. WHO Global report on hypertension 2025. Lancet. (2025) 406(10517):2318–9. 10.1016/S0140-6736(25)02208-1 41241506

[2] De La Sierra A. Blood pressure variability as a risk factor for cardiovascular disease: which antihypertensive agents are more effective? J Clin Med. (2023) 12(19):6167. 10.3390/jcm12196167 37834811 PMC10573370

[3] Wan EYF Yu EYT Chin WY Fong DYT Choi EPH Lam CLK. Association of visit-to-visit variability of systolic blood pressure with cardiovascular disease, chronic kidney disease and mortality in patients with hypertension. J Hypertens. (2020) 38(5):943–53. 10.1097/HJH.0000000000002347 31904623

[4] Liu Y Luo X Jia H Yu B. The effect of blood pressure variability on coronary atherosclerosis plaques. Front Cardiovasc Med. (2022) 9:803810. 10.3389/fcvm.2022.803810 35369353 PMC8965230

[5] Lattanzi S Słomka A Divani AA. Blood pressure variability and cerebrovascular reactivity. Am J Hypertens. (2023) 36(1):19–20. 10.1093/ajh/hpac114 36219582

[6] Karadag MF. A new potential risk factor for central serous chorioretinopathy: blood pressure variability. Eye. (2021) 35(8):2190–5. 10.1038/s41433-020-01222-1 33077907 PMC8302670

[7] Spallone V. Blood pressure variability and autonomic dysfunction. Curr Diab Rep. (2018) 18(12):137. 10.1007/s11892-018-1108-z 30361834

[8] Zhang Y Agnoletti D Blacher J Safar ME. Blood pressure variability in relation to autonomic nervous system dysregulation: the X-CELLENT study. Hypertens Res. (2012) 35(4):399–403. 10.1038/hr.2011.203 22129516

[9] Kishi T. Baroreflex failure and beat-to-beat blood pressure variation. Hypertens Res. (2018) 41(8):547–52. 10.1038/s41440-018-0056-y 29880837

[10] Parati G Bilo G Kollias A Pengo M Ochoa JE Castiglioni P Blood pressure variability: methodological aspects, clinical relevance and practical indications for management—a European society of hypertension position paper ∗. J Hypertens. (2023) 41(4):527–44. 10.1097/HJH.0000000000003363 36723481

[11] Schutte AE Kollias A Stergiou GS. Blood pressure and its variability: classic and novel measurement techniques. Nat Rev Cardiol. (2022) 19(10):643–54. 10.1038/s41569-022-00690-0 35440738 PMC9017082

[12] Clark D III Nicholls SJ St John J Elshazly MB Ahmed HM Khraishah H Visit-to-Visit blood pressure variability, coronary atheroma progression, and clinical outcomes. JAMA Cardiol. (2019) 4(5):437–43. 10.1001/jamacardio.2019.0751 30969323 PMC6537804

[13] Heshmatollah A Ma Y Fani L Koudstaal PJ Ikram MA Ikram MK. Visit-to-visit blood pressure variability and the risk of stroke in The Netherlands: a population-based cohort study. PLoS Med. (2022) 19(3):e1003942. 10.1371/journal.pmed.1003942 35298463 PMC8929650

[14] Steinsaltz D Patten H Bester D Rehkopf D. Short-Term and mid-term blood pressure variability and long-term mortality. Am J Cardiol. (2025) 234:71–8. 10.1016/j.amjcard.2024.10.005 39447722

[15] Bakkar NZ El-Yazbi AF Zouein FA Fares SA. Beat-to-beat blood pressure variability: an early predictor of disease and cardiovascular risk. J Hypertens. (2021) 39(5):830–45. 10.1097/HJH.0000000000002733 33399302

[16] Kulkarni S Parati G Bangalore S Bilo G Kim BJ Kario K Blood pressure variability: a review. J Hypertens. (2025) 43(6):929–38. 10.1097/HJH.0000000000003994 40084481 PMC12052075

[17] Ramirez AJ Parati G Castiglioni P Consalvo D Solis P R. Risk M Elderly hypertensive patients: silent white matter lesions, blood pressure variability, baroreflex impairment and cognitive deterioration. Curr Hypertens Rev. (2011) 7(2):80–7. 10.2174/157340211797457908

[18] Webb AJS Mazzucco S Li L Rothwell PM. Prognostic significance of blood pressure variability on beat-to-beat monitoring after transient ischemic attack and stroke. Stroke. (2018) 49(1):62–7. 10.1161/STROKEAHA.117.019107 29229726 PMC5742536

[19] Michard F Lopes MR Auler JO Jr. Pulse pressure variation: beyond the fluid management of patients with shock. Crit Care. (2007) 11(3):131. 10.1186/cc5905 17521454 PMC2206397

[20] Bojana S David M Nina J-Ž. The paraventricular nucleus of the hypothalamus in control of blood pressure and blood pressure variability. Front Physiol. (2022) 13:2022. 10.3389/fphys.2022.858941 PMC8966844 35370790

[21] Jia G Sowers JR. Hypertension in diabetes: an update of basic mechanisms and clinical disease. Hypertension. (2021) 78(5):1197–205. 10.1161/HYPERTENSIONAHA.121.17981 34601960 PMC8516748

[22] Courcelles L Stoenoiu M Haufroid V Lopez-Sublet M Boland L Wauthier L Laboratory testing for endocrine hypertension: current and future perspectives. Clin Chem. (2024) 70(5):709–26. 10.1093/clinchem/hvae022 38484135

[23] Lopes S Mesquita-Bastos J Garcia C Leitão C Ribau V Teixeira M Aerobic exercise improves central blood pressure and blood pressure variability among patients with resistant hypertension: results of the EnRicH trial. Hypertens Res. (2023) 46(6):1547–57. 10.1038/s41440-023-01229-7 36813985

[24] Seidel M Pagonas N Seibert FS Bauer F Rohn B Vlatsas S The differential impact of aerobic and isometric handgrip exercise on blood pressure variability and central aortic blood pressure. J Hypertens. (2021) 39(7):1269–73. 10.1097/HJH.0000000000002774 33470732

[25] Tomitani N Kanegae H Kario K. The effect of psychological stress and physical activity on ambulatory blood pressure variability detected by a multisensor ambulatory blood pressure monitoring device. Hypertens Res. (2023) 46(4):916–21. 10.1038/s41440-022-01123-8 36522422 PMC9754994

[26] Chang HC Wu CL Lee YH Gu Y-H Chen Y-T Tsai Y-W Impact of dietary intake of sodium and potassium on short-term blood pressure variability. J Hypertens. (2021) 39(9):1835–43. 10.1097/HJH.0000000000002856 34054053

[27] Narita K Hoshide S Kario K. Seasonal variation in day-by-day home blood pressure variability and effect on cardiovascular disease incidence. Hypertension. (2022) 79(9):2062–70. 10.1161/HYPERTENSIONAHA.122.19494 35770661

[28] Kim Y Mattos MK Esquivel JH Davis EM Logan J. Sleep and blood pressure variability: a systematic literature review. Heart Lung. (2024) 68:323–36. 10.1016/j.hrtlng.2024.08.016 39217647

[29] Gupta A Whiteley WN Godec T Rostamian S Ariti C Mackay J Legacy benefits of blood pressure treatment on cardiovascular events are primarily mediated by improved blood pressure variability: the ASCOT trial. Eur Heart J. (2024) 45(13):1159–69. 10.1093/eurheartj/ehad814 38291599 PMC10984564

[30] Chen H Zhang R Zheng Q Yan X Wu S Chen Y. Impact of body mass index on long-term blood pressure variability: a cross-sectional study in a cohort of Chinese adults. BMC Public Health. (2018) 18(1):1193. 10.1186/s12889-018-6083-4. 30348124 PMC6196453

[31] Schoina M Loutradis C Minopoulou I Theodorakopoulou M Pyrgidis N Tzanis G Ambulatory blood pressure trajectories and blood pressure variability in diabetic and non-diabetic chronic kidney disease. Am J Nephrol. (2020) 51(5):411–20. 10.1159/000507416 32259821

[32] Zhou TL Kroon AA Reesink KD Schram MT Koster A Schaper NC Blood pressure variability in individuals with and without (pre)diabetes: the Maastricht study. J Hypertens. (2018) 36(2):259–67. 10.1097/HJH.0000000000001543 28885385

[33] Lopacinska N Wesoly J Bluyssen HAR. Interplay between KLF4, STAT, IRF, and NF-*κ*B in VSMC and macrophage plasticity during vascular inflammation and atherosclerosis. Int J Mol Sci. (2025) 26(20):10205. 10.3390/ijms262010205 41155497 PMC12564505

[34] LiuS CaiJ ChenZ. Vascular mechanical forces and vascular diseases. J Adv Res. (2025):S2090-1232(25)00696-4 (article identifier) [Epub ahead of print]. 10.2991/978-94-6463-932-240975125

[35] Davis MJ Earley S Li YS Chien S. Vascular mechanotransduction. Physiol Rev. (2023) 103(2):1247–421. 10.1152/physrev.00053.2021 36603156 PMC9942936

[36] Ikeda Y Handa M Kawano K Kamata T Murata M Araki Y The role of von willebrand factor and fibrinogen in platelet aggregation under varying shear stress. J Clin Invest. (1991) 87(4):1234–40. 10.1172/JCI115124 2010539 PMC295144

[37] Gudayneh YA Shumye AF Gelaye AT Tegegn MT. Prevalence of hypertensive retinopathy and its associated factors among adult hypertensive patients attending at comprehensive specialized hospitals in northwest Ethiopia, 2024, a multicenter cross-sectional study. Int J Retina Vitreous. (2025) 11(1):17. 10.1186/s40942-025-00631-2 39962536 PMC11834681

[38] Tripathy K Modi P Arsiwalla T. Hypertensive Retinopathy. StatPearls. Treasure Island (FL): StatPearls Publishing LLC. (2025). 30252236

[39] Jiao L Lv C Zhang H. Effect of blood pressure variability on hypertensive retinopathy. Clin Exp Hypertens. (2023) 45(1):2205050. 10.1080/10641963.2023.2205050 37120839

[40] Lou Q Chen X Wang K Liu H Zhang Z Lee Y. The impact of systolic blood pressure, pulse pressure, and their variability on diabetes retinopathy among patients with type 2 diabetes. J Diabetes Res. (2022) 2022:7876786. 10.1155/2022/7876786 35359566 PMC8964233

[41] Fatulla P Ludvigsson J Imberg H Nyström T Lind M. Retinopathy and nephropathy in type 1 diabetes: role of HbA1c and blood pressure variability. Acta Diabetol. (2025) 62,(12,):2235–8. 10.1007/s00592-025-02575-3 40960629 PMC12727752

[42] Sohn MW Epstein N Huang ES Huo Z Emanuele N Stukenborg G Visit-to-visit systolic blood pressure variability and microvascular complications among patients with diabetes. J Diabetes Complications. (2017) 31(1):195–201. 10.1016/j.jdiacomp.2016.09.003 27671535 PMC5209256

[43] Foo V Quah J Cheung G Tan NC Ma Zar KL Chan CM Hba1c, systolic blood pressure variability and diabetic retinopathy in Asian type 2 diabetics. J Diabetes. (2017) 9(2):200–7. 10.1111/1753-0407.12403 27043025

[44] Cardoso CRL Leite NC Salles GF. Prognostic importance of visit-to-visit blood pressure variability for micro- and macrovascular outcomes in patients with type 2 diabetes: the Rio de Janeiro type 2 diabetes cohort study. Cardiovasc Diabetol. (2020) 19(1):50. 10.1186/s12933-020-01030-7 32359350 PMC7196231

[45] Dasa O Smith SM Howard G Cooper-DeHoff RM Gong Y Handberg E Association of 1-year blood pressure variability with long-term mortality among adults with coronary artery disease: a *post hoc* analysis of a randomized clinical trial. JAMA Netw Open. (2021) 4(4):e218418. 10.1001/jamanetworkopen.2021.8418 33914047 PMC8085725

[46] Sun PF Chen Y Zhan YQ Shen P-p Wu C-y Shen Y-b Association of 24-hour blood pressure average real variability with poor prognosis in critically ill patients with coronary artery disease. Sci Rep. (2025) 15(1):20676. 10.1038/s41598-025-08146-4 40596681 PMC12216912

[47] De La SA Mateu A Gorostidi M Vinyoles E Segura J Ruilope LM. Antihypertensive therapy and short-term blood pressure variability. J Hypertens. (2021) 39(2):349–55. 10.1097/HJH.0000000000002618 33031167

[48] Turana Y Tengkawan J Chia YC Nathaniel M Wang J Sukonthasarn A Hypertension and stroke in Asia: a comprehensive review from HOPE Asia. J Clin Hypertens (Greenwich). (2021) 23(3):513–21. 10.1111/jch.14099 33190399 PMC8029540

[49] Stamou E Iliakis P Konstantinidis D Manta E Kyriakoulis KG Kasiakogias A Association of blood pressure variability and incidence of stroke: a systematic review and meta-analysis. J Hypertens. (2025) 43(11):1764–72. 10.1097/HJH.0000000000004129 40986655

[50] Zhou TL Henry RMA Stehouwer CDA van Sloten TT Reesink KD Kroon AA. Blood pressure variability, arterial stiffness, and arterial remodeling. Hypertension. (2018) 72(4):1002–10. 10.1161/HYPERTENSIONAHA.118.11325 30354707

[51] Hoshide S Kario K. Morning surge in blood pressure and stroke events in a large modern ambulatory blood pressure monitoring cohort: results of the JAMP study. Hypertension. (2021) 78(3):894–6. 10.1161/HYPERTENSIONAHA.121.17547 34304583

[52] Webb AJS Werring DJ. New insights into cerebrovascular pathophysiology and hypertension. Stroke. (2022) 53(4):1054–64. 10.1161/STROKEAHA.121.035850 35255709 PMC7615037

[53] Ma Y Yilmaz P Bos D Blacker D Viswanathan A Ikram MA Blood pressure variation and subclinical brain disease. J Am Coll Cardiol. (2020) 75(19):2387–99. 10.1016/j.jacc.2020.03.043 32408975 PMC9049233

[54] Udani S Lazich I Bakris GL. Epidemiology of hypertensive kidney disease. Nat Rev Nephrol. (2011) 7(1):11–21. 10.1038/nrneph.2010.154 21079654

[55] Agarwal R. Hypertensive nephropathy: revisiting the causal link between hypertension and kidney disease. Nephrol Dial Transplant. (2025) 40(7):1270–2. 10.1093/ndt/gfaf014 39900493

[56] Cheng X Song C Ouyang F Ma T Fang F Zhang G Systolic blood pressure variability: risk of cardiovascular events, chronic kidney disease, dementia, and death. Eur Heart J. (2025) 46(27):2673–87. 10.1093/eurheartj/ehaf256 40249367 PMC12257294

[57] Sheikh AB Sobotka PA Garg I Dunn JP Minhas AMK Shandhi MMH Blood pressure variability in clinical practice: past, present and the future. J Am Heart Assoc. (2023) 12(9):e029297. 10.1161/JAHA.122.029297 37119077 PMC10227216

[58] Wang Z Li W Jiang C Hua C Tang Y Zhang H Association between blood pressure variability and risk of kidney function decline in hypertensive patients without chronic kidney disease: a *post hoc* analysis of systolic blood pressure intervention trial study. J Hypertens. (2024) 42(7):1203–11. 10.1097/HJH.0000000000003715 38690929

[59] Wang Y Zhao P Chu C Zhang X-Y Zou T Zhou H-W Associations of long-term visit-to-visit blood pressure variability with subclinical kidney damage and albuminuria in adulthood: a 30-year prospective cohort study. Hypertension. (2022) 79(6):1247–56. 10.1161/HYPERTENSIONAHA.121.18658 35360932 PMC9093226

[60] Lim S Chung SH Kim JH Joo HJ. Effects of metabolic parameters’ variability on cardiovascular outcomes in diabetic patients. Cardiovasc Diabetol. (2023) 22(1):114. 10.1186/s12933-023-01848-x 37189113 PMC10186656

[61] Chiriacò M Pateras K Virdis A Charakida M Kyriakopoulou D Nannipieri M Association between blood pressure variability, cardiovascular disease and mortality in type 2 diabetes: a systematic review and meta-analysis. Diabetes Obes Metab. (2019) 21(12):2587–98. 10.1111/dom.13828 31282073

[62] Wong YK Chan YH Hai JSH Lau K-K Tse H-F. Predictive value of visit-to-visit blood pressure variability for cardiovascular events in patients with coronary artery disease with and without diabetes mellitus. Cardiovasc Diabetol. (2021) 20(1):88. 10.1186/s12933-021-01280-z 33894788 PMC8070286

[63] Ikeda S Shinohara K Enzan N Matsushima S Tohyama T Funakoshi K Effectiveness of statin intensive therapy in type 2 diabetes mellitus with high visit-to-visit blood pressure variability. J Hypertens. (2021) 39(7):1435–43. 10.1097/HJH.0000000000002823 34001809

[64] Almuwaqqat Z Hui Q Liu C Zhou JJ Voight BF Ho Y-L Long-Term body mass Index variability and adverse cardiovascular outcomes. JAMA Netw Open. (2024) 7(3):e243062. 10.1001/jamanetworkopen.2024.3062 38512255 PMC10958234

[65] Koenen M Hill MA Cohen P Sowers JR. Obesity, adipose tissue and vascular dysfunction. Circ Res. (2021) 128(7):951–68. 10.1161/CIRCRESAHA.121.318093 33793327 PMC8026272

[66] Saxton SN Clark BJ Withers SB Eringa EC Heagerty AM. Mechanistic links between obesity, diabetes, and blood pressure: role of perivascular adipose tissue. Physiol Rev. (2019) 99(4):1701–63. 10.1152/physrev.00034.2018 31339053

[67] Li Y Liu Y Liu S Gao M Wang W Chen K Diabetic vascular diseases: molecular mechanisms and therapeutic strategies. Signal Transduct Target Ther. (2023) 8(1):152. 10.1038/s41392-023-01400-z 37037849 PMC10086073

[68] Deng Y Liu Y Zhang S Yu H Zeng X Chen Z Visit-to-visit variability of blood pressure and risk of macrovascular and microvascular complications in patients with type 2 diabetes: a Chinese primary-care cohort study. J Diabetes. (2022) 14(11):767–79. 10.1111/1753-0407.13331 36443961 PMC9705806

[69] Chen N Liu YH Hu LK Ma L-L Zhang Y Chu X Association of variability in metabolic parameters with the incidence of type 2 diabetes: evidence from a functional community cohort. Cardiovasc Diabetol. (2023) 22(1):183. 10.1186/s12933-023-01922-4 37474925 PMC10357611

[70] Kario K Ferdinand KC O'keefe JH. Control of 24-hour blood pressure with SGLT2 inhibitors to prevent cardiovascular disease. Prog Cardiovasc Dis. (2020) 63(3):249–62. 10.1016/j.pcad.2020.04.003 32275926

[71] Piché ME Tchernof A Després JP. Obesity phenotypes, diabetes, and cardiovascular diseases. Circ Res. (2020) 126(11):1477–500. 10.1161/CIRCRESAHA.120.316101 32437302

[72] Organization World Health. Global report on hypertension 2025: high stakes: turning evidence into action. Available online at: https://www.who.int/publications/i/item/9789240115569 (Accessed September 23, 2025).

[73] Tadic M Cuspidi C Pencic B Andric A Pavlovic SU Iracek O The interaction between blood pressure variability, obesity, and left ventricular mechanics: findings from the hypertensive population. J Hypertens. (2016) 34(4):772–80. 10.1097/HJH.0000000000000830 26825168

[74] Wang CX Kuang M Hou JJ Lin SY Liu SZ Bao N Association between obesity indicators and retinopathy in US adults: NHANES 2005−2008. Front Nutr. (2025) 12:11. 10.3389/fnut.2025.1598240 PMC12229866 40626226

[75] Sorop O Olver TD Van De Wouw J Heinonen I van Duin RW Duncker DJ The microcirculation: a key player in obesity-associated cardiovascular disease. Cardiovasc Res. (2017) 113(9):1035–45. 10.1093/cvr/cvx093 28482008

[76] Chen FY Lee CW Chen YJ Lin Y-H Yeh C-F Lin C-C Pathophysiology and blood pressure measurements of hypertension in the elderly. J Formos Med Assoc. (2025) 124(Suppl 1):S10–6. 10.1016/j.jfma.2025.03.027 40328594

[77] Rouch L Cestac P Sallerin B Piccoli M Benattar-Zibi L Bertin P Visit-to-Visit blood pressure variability is associated with cognitive decline and incident dementia: the S.AGES cohort. Hypertension. (2020) 76(4):1280–8. 10.1161/HYPERTENSIONAHA.119.14553 32862710

[78] Ernst ME Chowdhury EK Beilin LJ Margolis KL Nelson MR Wolfe R Long-Term blood pressure variability and risk of cardiovascular disease events among community-dwelling elderly. Hypertension. (2020) 76(6):1945–52. 10.1161/HYPERTENSIONAHA.120.16209 33131315 PMC7666049

[79] Ernst ME Fravel MA Webb KL Wetmore JB Wolfe R Chowdhury E Long-Term blood pressure variability and kidney function in participants of the ASPREE trial. Am J Hypertens. (2022) 35(2):173–81. 10.1093/ajh/hpab143 34519331 PMC8807162

[80] Wang DWM Rodrigues BCD Canziani ME Merli G Souza H Martins JdT Blood pressure variability in older patients with chronic kidney disease. Cardiorenal Med. (2025) 15(1):347–57. 10.1159/000545403 40239642

[81] Ram CVS. The triad of orthostatic hypotension, blood pressure variability, and arterial stiffness: a new syndrome? J Hypertens. (2020) 38(6):1031–2. 10.1097/HJH.0000000000002411 32371791

[82] Omboni S Kario K Bakris G Parati G. Effect of antihypertensive treatment on 24-h blood pressure variability: pooled individual data analysis of ambulatory blood pressure monitoring studies based on olmesartan mono or combination treatment. J Hypertens. (2018) 36(4):720–33. 10.1097/HJH.0000000000001608 29045341 PMC5862001

[83] De Havenon A Petersen N Wolcott Z Goldstein E Delic A Sheibani N Effect of dihydropyridine calcium channel blockers on blood pressure variability in the SPRINT trial: a treatment effects approach. J Hypertens. (2022) 40(3):462–9. 10.1097/HJH.0000000000003033 34694261 PMC11284837

[84] Fan JL O'donnell T Lanford J O’Donnell T Croft K Watson E Dietary nitrate reduces blood pressure and cerebral artery velocity fluctuations and improves cerebral autoregulation in transient ischemic attack patients. J Appl Physiol (1985). (2020) 129(3):547–57. 10.1152/japplphysiol.00160.2020 32758038

[85] Hao Z Tran J Lam A Yiu K Tsoi K. Aerobic, resistance, and isometric exercise to reduce blood pressure variability: a network meta-analysis of 15 clinical trials. J Clin Hypertens (Greenwich). (2025) 27(5):e70050. 10.1111/jch.70050 40326294 PMC12053447

[86] Caminiti G Iellamo F Mancuso A Cerrito A Montano M Manzi V Effects of 12 weeks of aerobic versus combined aerobic plus resistance exercise training on short-term blood pressure variability in patients with hypertension. J Appl Physiol (1985). (2021) 130(4):1085–92. 10.1152/japplphysiol.00910.2020 33630677

[87] Schiavon CA Ikeoka D Santucci EV Santos RN Damiani LP Bueno PT Effects of bariatric surgery versus medical therapy on the 24-hour ambulatory blood pressure and the prevalence of resistant hypertension. Hypertension. (2019) 73(3):571–7. 10.1161/HYPERTENSIONAHA.118.12290 30661477

